# A forgotten aspect of the NICE reference case: an observational study of the health related quality of life impact on caregivers of people with multiple sclerosis

**DOI:** 10.1186/1472-6963-13-346

**Published:** 2013-09-09

**Authors:** Sarah Acaster, Rodolphe Perard, Deven Chauhan, Andrew J Lloyd

**Affiliations:** 1 Oxford Outcomes Ltd, Seacourt Tower, West Way, Oxford, UK; 2 Merck Serono Ltd, Bedfont Cross, Stanwell Road, Feltham, Middlesex, UK

**Keywords:** Health related quality of life, Burden of illness, Multiple sclerosis, Disease severity, Indirect costs

## Abstract

**Background:**

There are few published health technology assessments that have included the impact of a disease or treatment on caregivers’ health related quality of life (HRQL). The objectives of this study were to explore the overall HRQL of caregivers of people with Multiple Sclerosis compared to matched controls, and more specifically explore the impact of different levels of functioning in people with MS on caregivers’ HRQL scores.

**Methods:**

A cross sectional observational study conducted as an online survey was undertaken in the UK. 200 caregivers of people with MS and 200 matched controls completed the EQ-5D, SF-36, HADS and a socio-demographic questionnaire. Caregivers also completed the Patient Determined Disease Steps questionnaire as a measure of MS severity. Differences in HRQL between caregivers and controls were assessed using t-tests and chi square analyses as appropriate. Ordinary Least Squares regression was also used to evaluate the disutility of being a caregiver compared to controls stratified by MS severity.

**Results:**

Caregivers reported significantly lower HRQL, as measured by the SF-36, HADS and EQ-5D, compared to matched controls. A lower level of functioning in people with MS was mirrored by lower levels of caregivers’ HRQL. At the point at which mobility problems required wheelchair use caregivers reported better HRQL.

**Conclusions:**

The potential impact of caregiving on HRQL is an important consideration for economic evaluations. In relation to MS, the results suggest that caregiver’s HRQL deteriorates as the people with MS lose functioning; thus treatments that delay loss of function may have a benefit for caregivers as well as patients.

## Background

Caring for partners, dependents or relatives with chronic disease can have a substantial impact on the health of the caregiver [1-3]. This has been recognised by policy makers and the UK Department of Health (DH). A standing commission on caregivers was established in 2009 and the DH has been promoting a national strategy for caregivers since 1999. The physical and emotional impact associated with being a caregiver is also recognised by the National Institute for Health and Clinical Excellence (NICE) in their review of the cost effectiveness of new therapies. The NICE methods guide (2013) for manufacturers’ highlights how the indirect impact of a disease or treatment on caregivers can be considered when assessing the cost effectiveness of therapies. A treatment that maintains or improves a caregivers’ health related quality of life (HRQL) is a relevant benefit to be considered in an economic evaluation. NICE use relevant HRQL data (in the form of a utility) to estimate quality adjusted life years in cost utility analysis and has a preference for the EQ-5D [4].

Despite this, the impact of caregiving on health has infrequently been considered in health technology assessments (HTA) in the UK. Two reviews of outcomes data included in NICE appraisals [5,6] make no reference to any appraisals that directly included caregiver HRQL data. Two appraisals (for Alzheimer’s disease and Multiple Sclerosis) included adjustments to mean utility values to represent the effect of treatment on carers HRQL, and both utilized the same Alzheimer’s caregiver data. From an economic evaluation perspective this seems to be an under researched area [7-9]. However, given the high prevalence of unpaid caregivers in a range of therapy areas we believe more work is needed. Further discussion of the role and importance of caregiving has recently been called for [10].

The present study aimed to measure the impact on HRQL of being an unpaid caregiver of someone with Multiple Sclerosis (MS) including the EQ-5D as the measure preferred by NICE for economic evaluations. Previous research has demonstrated the negative impact on HRQL of being a caregiver to a person with MS, with added burden associated with increased disease severity [11-14]. However, to the authors’ knowledge, no exploration of the potential for this impact to be captured by preference based utility measures suitable for economic analysis, such as the EQ-5D, has been conducted to date. Thus, the aim of the present study was to explore the overall HRQL of caregivers of people with MS including a utility measure compared to matched controls, and more specifically explore the impact of different levels of functioning in people with MS and how that related to caregiver’s HRQL compared to matched controls. This study was exploratory and was designed as a cross sectional survey of caregivers of people with MS and a matched control sample from the UK general population.

## Methods

### Design and participants

A cross sectional observational study conducted as an online survey was undertaken in the UK. 200 caregivers and 200 controls, matched on age, sex, employment status and habitation status, were recruited from the general population using a specialist patient recruitment panel. Response rates were 75% for caregivers (200/266) and 95% for the general population (200/211). All caregivers reported providing unpaid care to a person with MS on a daily basis, information regarding the type of activities and time spent daily providing care were collected for descriptive purposes but not utilised for screening purposes or statistical analyses. All controls were screened to ensure they did not provide unpaid care to an individual \with any disease or disorder.

### Ethics

Independent ethical review was sought and granted by Schulman Associates Institutional Review Board Inc. Informed written consent was obtained from all participants prior to completion of the online survey.

### Survey

Potential participants were sent an email asking if they would be interested in participating in a survey that would involve answering some questions about themselves (and the person they cared for, if a caregiver). Interested participants followed a link provided in the email to get to the consent form, screening questions, and the survey. Caregivers were recruited first, followed by the general population control group, in order to match the two groups on the socio-demographic variables detailed above. The survey was conducted in March 2010.

The HRQL measures included the EQ-5D [15,16]; the SF-36 [17], and the Hospital Anxiety and Depression Scale (HADS; [18]). Caregivers only were also asked to complete the Patient Determined Disease Steps Scale (PDDS; [19-22]). The PDDS is a self-complete tool which is designed to provide an assessment of functional loss in MS. It was used here to stratify the disease severity of the people the caregivers were providing care for. All participants also completed a socio-demographic form. Each of these measures is described in more detail below.

The EQ-5D is a generic health status measure. Participants indicate their current health status on five domains (mobility; self-care; usual activity; pain/discomfort; anxiety/depression) as either no problems, some problems or severe problems. Participants also indicate their current health on a visual analogue scale. Health utilities were derived from the EQ-5D using UK general population preference weights [23], which provide a potential range of scores from - 0.59 to 1.0 (where a score of 1 represents full health and a score of 0 represents dead).

The SF-36 Version 2 (4 week recall period) is a generic HRQL instrument. It is intended for use in a wide range of conditions as well as the general population, and can be self-administered. There are 36 items that assess health across 8 domains: bodily pain, general health perceptions, mental health, physical functioning, role limitations due to emotional health problems, role limitations due to physical health problems, social functioning, and vitality. All items use categorical response options (range: 2–6 options). Two component summary scores for physical and mental health can also be calculated. The scores for each subscale are converted to norm based scores (based on 1998 US general population), with a mean of 50 and a standard deviation of 10. A score of 100 represents the best health.

The HADS is a self-administered measure of anxiety and depression. It has 14 items divided equally between two subscales; the HADS-A (Anxiety subscale) and the HADS-D (Depression subscale). Each item is rated on a 4-point scale (0 = not at all, to 3 = yes definitely), thus each subscale has a score ranging from 0–21. Research suggests that ‘caseness’ (clinical mood disturbance) is defined by a cut off score of ≥ 8 [24].

The PDDS is a self-assessment scale that assesses functional disability in people with MS. It is based on the earlier clinician rated Disease Steps Scale (DSS) developed by [19], which maps to the Expanded Disability Severity Scale, EDSS [25]. In line with the EDSS, the PDDS is a 9 point scale focusing mainly on mobility: 0 (normal) to 8 (bedridden; see Table 1 for a detailed description of each level). The wording of the PDDS was slightly modified, with the developer’s permission, from a patient reported instrument to a caregiver proxy instrument for the purposes of this study.

**Table 1:** Patient determined disease steps

**0**	**Normal:** The person that I care for may have some mild symptoms, mostly sensory due to MS but these do not limit his/her activity. If he/she does have an attack, he/she returns to normal when the attack has passed.
**1**	**Mild Disability:** The person that I care for has some noticeable symptoms from his/her MS but they are minor and have only a small effect on his/her lifestyle.
**2**	**Moderate Disability:** The person that I care for doesn’t have any limitations in his/her walking ability. However, he/she does have significant problems due to MS that limit daily activities in other ways.
**3**	**Gait Disability:** MS does interfere with his/her activities, especially his/her walking. He/she can work a full day, but athletic or physically demanding activities are more difficult than they used to be. He/she usually doesn’t need a cane or other assistance to walk, but he/she might need some assistance during an attack.
**4**	**Early Cane:** The person that I care for uses a cane or a single crutch or some other form of support (such as touching a wall or leaning on someone’s arm) for walking all the time or part of the time, especially when walking outside. I think he/she can walk 25 feet in 20 seconds without a cane or crutch. He/she always needs some assistance (cane or crutch) if he/she wants to walk as far as 3 blocks.
**5**	**Late Cane:** To be able to walk 25 feet, the person that I care for has to have a cane, crutch or someone to hold onto. He/she can get around the house or other buildings by holding onto furniture or touching the walls for support. He/she may use a scooter or wheelchair if he/she wants to go greater distances.
**6**	**Bilateral Support:** To be able to walk as far as 25 feet the person that I care for must have 2 canes or crutches or a walker. He/she may use a scooter or wheelchair for longer distances.
**7**	**Wheelchair/Scooter:** His/her main form of mobility is a wheelchair. He/she may be able to stand and/or take one or two steps, but he/she can’t walk 25 feet, even with crutches or a walker.
**8**	**Bedridden:** The person that I care for is unable to sit in a wheelchair for more than one hour.

The socio-demographic form included questions related to age, gender, ethnicity, educational level, employment status, living situation and whether they suffer from any illnesses. The caregivers were also asked questions about their role as a caregiver and the person they care for, for example, how long they have been a caregiver, their relationship to the person with MS, how many hours of care they provide per day, what type of MS the person they care for has, what type of activities they help with, and whether they get any support themselves.

### Statistical analysis

The caregiver and control groups’ socio-demographic details were tabulated and analysed for any significant between group differences using t-tests or chi square analyses as appropriate. The SF-36 and HADS questionnaires were scored according to their published scoring instructions. Any potential HRQL differences between caregivers and controls, as measured by these instruments, were assessed using independent t-tests, effect size calculations and chi square analyses as appropriate.

Health utilities were derived from the EQ-5D using validated scoring instructions and UK general population weightings [23]. The difference in EQ-5D utility values between groups was assessed using an independent t-test. Ordinary least squares regression was used to further examine the group differences with the caregiver population stratified by MS severity, as measured by the PDDS. Eight groups were created (controls and PDDS 0–1; 2–3; 4; 5; 6; 7; 8) and dummy coded with the controls as the reference category [26]. PDDS groups 0 and 1, and 2 and 3 were collapsed due to small sample sizes in individual categories (n < 10). Socio-demographic variables were included in the model as covariates, as the caregivers and controls were only matched at the group level; thus creating variability once the caregiver sample was stratified by MS severity. All analyses were conducted in SPSS version 18.

## Results

The socio-demographic data for the control and caregiver samples are presented in Table 2. In line with the sampling methodology there were no significant differences in age, gender, education, employment or living status between groups. Similarly, there were no group differences in the proportion of people reporting a number of common diseases or disorders: heart disease, arthritis, diabetes or cancer. However, there were significantly higher incidences of self-reported psychological problems amongst the caregiver population compared to controls: stress (***χ***2 = 41.0, p < .001), anxiety (***χ***2 = 20.8, p < .001), depression (***χ***2 = 11.1, p < .01), fatigue (***χ***2 = 45.3, p < .001), and sleep problems (***χ***2 = 17.8, p < .001).

**Table 2:** Socio-demographic descriptive statistics for control and caregiver groups

**Characteristic**			**Controls**	**Caregivers**
N			200	200
Age		Mean (SD)	50.99 (13.80)	50.88 (13.48)
Gender	Male	N (%)	106 (53.0%)	109 (54.5%)
Ethnicity	Caucasian	N (%)	189 (94.5%)	186 (93.0%)
Education	Secondary school	N (%)	95 (47.5%)	99 (49.5%)
	University		70 (35.0%)	66 (33.0%)
	Other		35 (17.5%)	35 (17.5%)
Employment	Employed full-time	N (%)	66 (33.0%)	63 (31.5%)
	Employed part-time		28 (14.0%)	29 (14.5%)
	Retired		62 (31.0%)	64 (32.0%)
	Other		44 (22.0%)	44 (22.0%)
Living Status	Living with partner	N (%)	179 (89.5%)	181 (90.5%)
	Living alone		6 (3.0%)	6 (3.0%)
	Other		15 (7.5%)	13 (6.5%)
Self-reported Illness/Conditions	Stress	N (%)	44 (22.0%)	106 (53.0%)
	Anxiety		32 (16.0%)	72 (36.0%)
	Depression		32 (16.0%)	60 (30.0%)
	Fatigue		37 (18.5%)	101 (50.5%)
	Sleep problems		48 (24.0%)	88 (44.0%)
	Heart disease		11 (5.5%)	13 (6.5%)
	Arthritis		44 (22.0%)	33 (16.5%)
	Diabetes		17 (8.5%)	21 (10.5%)
	Cancer		5 (2.5%)	3 (1.5%)

N = Sample Size; SD = Standard Deviation.

Background characteristics of the MS caregivers and patients they care for revealed considerable variability (Table 3). The majority of caregivers provided assistance with various activities of daily living and care for 5–24 hours per day, and received no formal support.

**Table 3:** Caregiver and caregiver-reported MS characteristics

**Characteristic**			**Distribution**
Length of time as caregiver (years)		Mean (SD)	9.57 (8.47)
Length of time individual has had MS (years)		Mean (SD)	14.93 (12.83)
Type of MS	Relapsing Remitting MS (RRMS)	N (%)	50 (25.0%)
	Secondary Progressive MS (SPMS)		87 (43.5%)
	Primary Progressive MS (PPMS)		63 (31.5%)
PDDS Level*	0	N (%)	12 (6.0%)
	1		16 (8.0%)
	2		15 (7.5%)
	3		9 (4.5%)
	4		20 (10.0%)
	5		26 (13.0%)
	6		27 (13.5%)
	7		62 (31.0%)
	8		13 (6.5%)
Relationship between caregiver and individual with MS	Partner/Spouse	N (%)	153 (76.5%)
	Parent		13 (6.5%)
	Child		4 (2.0%)
	Family Other		19 (9.5%)
	Friend		11 (5.5%)
Live with the person you provide care for?	Yes	N (%)	168 (84.0%)
Number of hours a day providing care	1 - 4	N (%)	55 (27.5%)
	5 - 9		46 (23.0%)
	10 - 24		99 (49.5%)
Help with activities of daily living	Getting in/out of bed	N (%)	116 (58.0%)
	Dressing		116 (58.0%)
	Washing		102 (51.0%)
	Cooking		157 (78.5%)
	Eating		59 (29.5%)
	Getting around inside the home		104 (52.0%)
	Getting around outside the home		156 (78.0%)
Help with caregiving?	None	N	93
	Friends/Relatives		85
	External formal care giver		51

N = Sample Size; SD = Standard Deviation; * See Table 1 for description of PDDS levels.

**Figure 1:**
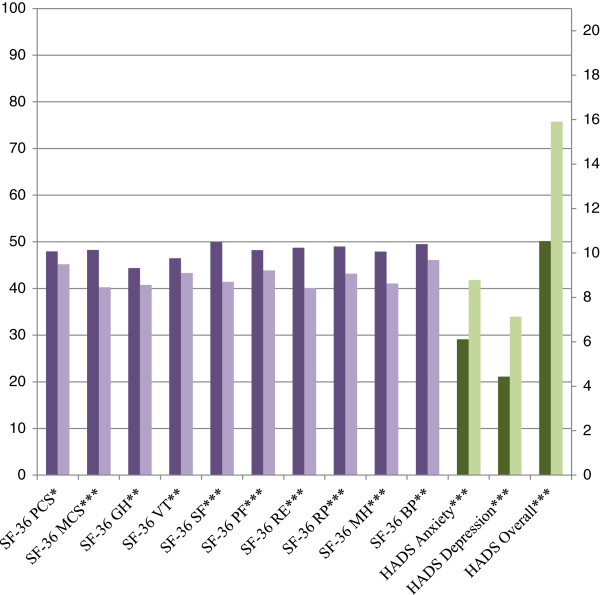
**SF-36 and HADS descriptive statistics and between groups analysis.** (royal purple bar) SF – 36 control mean, (lavender bar) SF – 36 caregiver mean, (dark olive green bar) HADS control mean, and (pistachio bar) HADS caregiver mean. PCS = physical component summary, MCS = mental component summary, GH = general health domain, VT = vitality domain, SF = social functioning domain, PF = physical functioning domain, RE = role emotional, RP = role physical, MH = mental health domain, BP = bodily pain domain. *p < 0.05; **p < 0.01; ***p < 0.001.

As shown in Figure 1, the MS caregivers’ health related quality of life was lower than the socio-demographically matched controls on all domains of the SF-36. The group comparisons were statistically significant across all domains and summary scores, with the greatest differences seen in relation to mental health and physical and social functioning. Similarly, the HADS results showed that caregivers of people with MS reported significantly higher levels of anxiety and depression than controls. Furthermore in terms of probable clinical ‘caseness’ , the HADS scores demonstrated that, the caregiver population contained a significantly greater proportion of probable anxiety ‘cases’ (***χ***2 = 38.83, p < .001) and depression cases (***χ***2 = 25.71, p < .001). The frequency of anxiety and depression probable clinical cases by population is presented in Table 4.

**Table 4:** Frequency of HADS anxiety and depression ‘cases’ by study population

	**Controls**		**Caregivers**	
	n	(%)	n	(%)
HADS Anxiety Probable Clinical Case*	59	(29.5%)	121	(60.5%)
HADS Depression Probable Clinical Case*	39	(19.5%)	86	(43.0%)

*Score ≥ 8 = probable clinical case [24].

**Figure 2:**
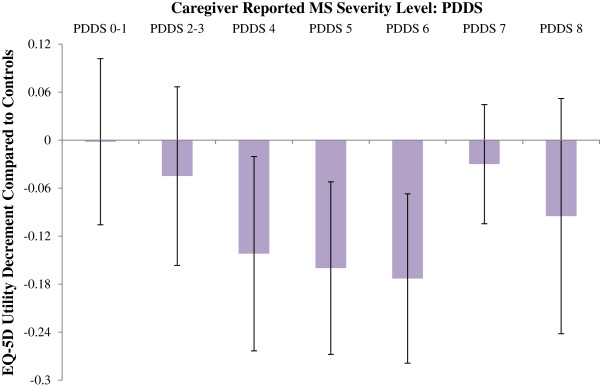
EQ-5D utility decrement associated with caregivers compared to controls by MS severity level (error bars represent 1.96 × SE).

The EQ-5D utility values also demonstrated a significant difference between the controls and caregivers of people with MS: M = 0.82, SD = 0.25 and M = 0.74, SD = 0.28, respectively [t (398) = 2.99, p = .003]. The estimated disutility values associated with being a caregiver relative to the control population, stratified by PDDS MS severity and with all socio-demographic variables considered as covariates, are presented in Figure 2. These show lower caregiver utility compared to controls in line with greater loss of functioning in people with MS. At PDDS 0–1 the difference between caregivers and controls is negligible (− 0.002, SE = 0.053), increasing at PDDS 2 – 3 (− 0.045, SE = 0.057) and showing significant differences between caregivers and controls at PDDS levels 4, 5 and 6, [disutility - 0.142 (SE = 0.062), - 0.160 (SE = 0.055), - 0.173 (SE = 0.054), respectively]. At PDDS level 7 (a level where the person with MS would typically require use of a wheelchair) the caregiver’s quality of life is less affected (PDDS 7: - 0.030, SE = 0.038) and appears in line with disutility at PDDS 2 – 3. This is also true at PDDS 8 where the person with MS would be predominantly bedridden (PDDS 8: - 0.095, SE = 0.075). As illustrated by the error bars in Figure 2 (+ / - 1.96 * SE), only PDDS groups 4, 5 and 6 demonstrated significantly lower utility scores than the control population.

Post hoc analyses were conducted to explore the reduced disutility at PDDS 7 and 8 by evaluating the impact of receiving external caregiver support including professional assistance from social services. No difference in utility values were found for caregivers recei ving versus not receiving support (M = 0.76, SD = 0.23 and M = 0.77, SD = 0.22, respectively).

## Discussion

This study aimed to explore the HRQL of caregivers of people with MS compared to matched controls. There was a particular interest in the impact of disease severity on the disutility associated with being a caregiver compared to controls, due to the paucity of caregiver data currently being considered in economic analyses. The results demonstrated that caregivers of people with MS reported significantly lower HRQL, as measured by the SF-36, HADS and EQ-5D, compared to matched controls. There was also a significantly greater proportion of HADS anxiety and depression clinical cases amongst caregivers compared to matched controls. Furthermore, lower levels of functioning in people with MS was mirrored by lower levels of caregiver’s HRQL up until the point at which mobility problems required wheelchair use. However, once caregiver reported MS severity had progressed to this point the difference in health utility scores between caregivers and controls reduced, indicative of better caregiver HRQL at the most advanced levels of MS severity. The reason for this is unclear and while post hoc analyses suggested that this was not due to reduced burden from external caregiver support, this and other potential explanations could be usefully further researched in a longitudinal study.

The significant impact of providing care on HRQL demonstrated here provides support for the need to consider caregivers’ HRQL in economic assessments of new health technologies. Furthermore, contrary to concerns expressed by Al-Janabi et al. [7], the results also suggest that in line with the NICE reference case the EQ-5D may be an appropriate measure to capture caregiver HRQL (2013). However, we also believe that there may be merit in conducting further research into the development of a caregiver specific measure to capture their specific concerns. The high level of variability seen in the EQ-5D utility scores at each PDDS level suggests that core concepts associated with being a caregiver to a person with MS may not currently be captured by the EQ-5D, and this could be explored through qualitative research. For example, the pattern of disutility captured by the EQ-5D here suggests the potential physical and psychological impact associated with caring for an individual who is bedridden may not be captured by the EQ-5D. Another potential explanation for the reduced disutility observed with increased disease severity found here may be the development of coping strategies or changes in health/disease perception over time. A different study design would be needed to address this hypothesis properly.

The results from the current study reflect a different pattern of HRQL impact than the values used in the NICE HTA submission in MS that utilised caregiver data [6]. That assessment assumed significant disutility only occurred at the very severe stages of MS; an assumption based on data from caregivers of people with Alzheimer’s disease. While the current findings are exploratory and need to be considered in light of the study limitations and the potential limitations of the EQ-5D to assess HRQL in caregivers, they do suggest that caregiver HRQL is impacted earlier in disease progression than previously indicated. Thus, caregiver utility data may be an important consideration in the evaluation of new MS treatments associated with slowing MS progression from relatively early stages of disease.

### Limitations

Given the exploratory nature of this study there are some limitations that need to be considered. Primarily, this was a cross sectional study of a progressive disease, and ideally the disutility of providing care should be measured longitudinally in order to capture potential within person changes over time. Additionally, due to the cross sectional nature of the study there were also uneven numbers in the different PDDS groups, with particularly small sample sizes in PDDS 8 for the stratified analysis. Variability in patient symptoms, and therefore the exact type of care provided, beyond those captured by the PDDS were also not considered in the stratified PDDS analysis and these may have a direct impact on the caregivers HRQL. Finally, a proxy version of the PDDS was used, with the caregivers reporting on their perception of the MS severity of the person they provide care for rather than the people with MS themselves. This caregiver reported version of the PDDS has not been correlated with the patient-reported PDDS and therefore may be a source of error if the utility values presented here are applied to patient reported data. However, given the concrete nature of the PDDS classifications this is likely to have a minimal impact.

## Conclusions

This study has provided evidence of the significant negative impact caregiving can have on HRQL and supports the potential consideration of caregiver HRQL in economic assessments of new treatments. The results also suggest that the EQ-5D is an appropriate measure of caregiver HRQL, but given the exploratory nature of the study and the pattern of disutility associated with MS severity reported here; further research into the measurement of caregiver HRQL is also warranted. In relation to MS, the findings reported here suggest the impact of disease progression on caregiver HRQL should be considered relatively early when evaluating new treatments. Issues relating to exactly how caregiver disutility should be incorporated into economic assessments and consideration of other pertinent issues, such as cultural differences related to caregiving, also require further consideration.

## Competing interests

The work was funded by Merck Serono and conducted by Oxford Outcomes, part of ICON plc. RP holds stock and/or stock options in Merck Serono through his employment. All other authors declare that they have no competing interests.

## Authors’ contributions

SA helped to conceive, design, and undertake the study. She also performed the statistical analysis, interpreted the data and drafted the manuscript. RP conceptualised the need for the study and helped interpret the data and findings and critically reviewed the manuscript. DC also conceptualised the need for the study and helped interpret the data and findings and provided some of the policy context for the manuscript. AJL also helped to conceive and design the study and wrote sections of the manuscript. All authors have read and approved the final manuscript.

## Pre-publication history

The pre-publication history for this paper can be accessed here:

http://www.biomedcentral.com/1472-6963/13/346/prepub
